# Pain management in people with severe mental illness: an agenda for progress

**DOI:** 10.1097/j.pain.0000000000002633

**Published:** 2022-03-16

**Authors:** Juliana Onwumere, Brendon Stubbs, Mary Stirling, David Shiers, Fiona Gaughran, Andrew S.C. Rice, Amanda C de C Williams, Whitney Scott

**Affiliations:** a Department of Psychology, Institute of Psychiatry, Psychology and Neuroscience, King's College London, London, United Kingdom; b National Psychosis Service, South London and Maudsley NHS Foundation Trust, Bethlem Royal Hospital, United Kingdom; c Department of Psychological Medicine, Institute of Psychiatry, Psychology and Neuroscience, King's College London, London, United Kingdom; d Physiotherapy Department, South London and Maudsley NHS Foundation Trust, London, United Kingdom; e Involvement Register Member of South London and Maudsley NHS Foundation Trust, London, United Kingdom; f Service User Member of Oxleas NHS Foundation Trust, London, United Kingdom; g Mind and Body Expert Advisory Group, King's Health Partners, London, United Kingdom; h Patient Governor of Guy's and St Thomas' NHS Foundation Trust, London, United Kingdom; i Psychosis Research Unit, Greater Manchester Mental Health NHS Trust, Manchester, United Kingdom; j Division of Psychology and Mental Health, University of Manchester, Manchester, United Kingdom; k Primary Care and Health Sciences, Keele University, Keele, United Kingdom; l Department of Psychosis Studies, Institute of Psychiatry, Psychology and Neuroscience, King's College London, London, United Kingdom; m Pain Research Group, Department of Surgery & Cancer, Faculty of Medicine, Imperial College London, London, United Kingdom; n Research Department of Clinical, Educational, and Health Psychology, University College London, London, United Kingdom; o Health Psychology Section, Department of Psychology, Institute of Psychiatry, Psychology and Neuroscience, King's College London, London, United Kingdom; p INPUT Pain Management Unit, Guy's and St Thomas' NHS Foundation Trust, London, United Kingdom

## Abstract

Supplemental Digital Content is Available in the Text.

## 1. Prevalence and impact of pain in people with severe mental illness

There is growing recognition that people with severe mental illness (SMI) have substantially poorer physical health and die 15 to 20 years prematurely, largely because of poor physical health.^30^ Severe mental illness often refers to schizophrenia spectrum (psychosis), bipolar, and major depressive disorders, following World Health Organization (WHO) and World Psychiatric Association terminology.^21,39^ The global prevalence of schizophrenia is 0.28% (95% uncertainty interval: 0.24-0.31),^16^ of bipolar disorder is 0.7% (0.6-0.8),^28^ and of major depression disorders is 4.4% (4.1-4.7).^27^ Severe mental illness is associated with increased likelihood of physical health problems (eg, diabetes and HIV)^30^ with a high burden of pain.^1,49^

Although the pain–depression relationship has been studied extensively, innovations are still needed to optimize pain management in people with comorbid major depression.^6,48^ Pain management in people with bipolar and psychotic disorders is underresearched.^106,109^ This review will highlight diagnostically specific challenges for pain management across major depression, bipolar disorder, and psychosis and discuss methodological approaches that may prove useful for improving pain management across them. This review does not capture other mental disorders that are often comorbid with and complicate pain management. These include anxiety,^3^ posttraumatic stress,^32^ substance use,^73^ and personality disorders,^23,51^ which are sometimes categorised with SMI when they cause severe functional impairment.^88^ Given the brevity of this review, we have chosen the narrower SMI definition to begin to advance this discussion.

Depression and pain commonly co-occur, although estimates of pain prevalence in people with depression vary widely (15%-100%; mean 65%).^6^ Pain prevalence is also high in people with bipolar disorder: 1 meta-analysis (k = 22) found that 29% of people with bipolar disorder (n = 171,352) reported pain, over double the risk of healthy controls (n = 12,342,577).^106^ Some doubt arises from unvalidated pain assessments used by many studies in the review.^106^

The pain–psychosis association is complex. Meta-analyses of experimental studies show that adults with schizophrenia have elevated pain thresholds and tolerance, independent of antipsychotic medication.^110^ However, a meta-analysis (k = 14) found that 35% of people with schizophrenia (n = 242,703) reported clinical pain, similar to age-matched and sex-matched controls without mental illness (n = 4,259,221).^109^ Given the heightened cardiometabolic burden in psychosis^35^ and the association between pain and cardiometabolic risk factors, such as obesity^37^ and diabetes, this raises the possibility that pain may be underrecognized or underreported in this group.^109^ Adding to this complexity, in 1 study of people with psychosis (n = 438), comorbid depressive symptoms were associated with clinically relevant pain.^107^ Therefore, pain reporting may be diagnostically specific, with psychosis associated with underreporting and (comorbid) depression with increased reporting.

As in the general population, pain is associated with increased disability and poorer quality of life in people with SMI.^70,107^ Importantly, both SMI and pain are associated with increased suicide risk.^34,89^ However, pain is not routinely assessed and managed in this population,^107^ and pain communication and assessment might be obscured by the nature of the SMI. Among people with psychosis, reduced self- and clinician-identification of pain may partially explain under or late recognition of conditions that commonly present with pain, such as appendicitis and cardiovascular disease; this may also partially explain the higher rates of late, inadequate, or absent treatment, adding to excess morbidity and mortality.^19,30,103,109^ Routine assessment and management of pain and its causes needs to improve in this population. There is also urgency to find ways to identify acute time-sensitive conditions earlier if pain is not serving as an indicator.

## 2. Understanding pain and its treatment in people with severe mental illness: where is the evidence?

Understanding of biopsychosocial^26,67,117^ influences on pain and related disability in people with SMI, particularly those with psychosis and bipolar disorder, is scarce and may be diagnostically specific. Research is needed to reconcile comparable rates of reported clinical pain in people with psychosis and healthy controls with mechanisms underpinning reduced experimental pain sensitivity.^109,110^ Increased striatal dopamine^46^ may contribute to pain underreporting in this population, given converse evidence of a negative association between striatal dopamine and pain in Parkinson disease.^92^ By contrast, serotonin and norepinephrine depletion may account for the depression–pain link.^36^ Of course, mechanisms explaining differences in pain reporting in SMI are likely multifactorial. Presently, research is lacking to understand whether and how positive symptoms (eg, delusions and hallucinations), negative symptoms (eg, poverty of speech), and thought disorder influence the meaning attributed to pain and related behavioral responses. One qualitative study of veterans with bipolar disorder and persistent pain described their sense of disconnecting from pain and overactivity during manic episodes, which increases pain.^114^ However, investigation of variations in the experience, impact, and communication of pain associated with different SMI features (eg, negative symptoms or depressive comorbidities in schizophrenia and mania in bipolar disorder) is in its infancy.^107,110,114^

Understanding of pain treatments in people with SMI is also limited. Recommendations for managing persistent pain include exercise, psychological therapy, and analgesic optimisation (where indicated).^77,78^ However, to increase sample homogeneity, people with SMI are often excluded from randomized controlled trials (RCTs) of pain treatments. Evidence is thus needed to understand the applicability of existing pain treatments to this population.

Psychological and exercise-based treatments are often combined to reduce pain-related distress and disability. Meta-analyses of RCTs show that cognitive-behavioral therapy for pain produces small- to medium-sized improvements in disability and mood compared with treatment as usual.^118^ However, little is known about the efficacy of psychologically informed interdisciplinary pain management for people with SMI because they are often excluded from trials. Of 75 trials in the 2020 Cochrane review of psychological therapies for pain,^118^ 60% explicitly excluded people with SMI (Table 1). Psychosis or schizophrenia was the most common exclusion (35% of studies), followed by any serious psychiatric disorder (23%). Of the remaining trials, only 1 reported the sample proportion with comorbid SMI, so we know very little about the efficacy of psychological treatments for pain in this population. This is echoed in clinical practice. For example, an audit from a large pain clinic in England indicated complex mental health needs (eg, severe depression) as one of the most common exclusion reasons from interdisciplinary treatment or short-term individual psychological therapy for pain.^58^

**Table 1:** Severe mental illness exclusion criteria listed in randomized controlled trials included in the most recent Cochrane review of psychological therapies for chronic pain.

SMI exclusion criteria	Frequency (%) of trials reporting the exclusion*
Psychosis/schizophrenia†	26 (34.7%)
Any serious psychiatric or psychological disorder†	17 (22.7%)
Suicide risk	10 (13.3%)
Bipolar disorder†	7 (9.3%)
Severe/significant depression	6 (8.0%)
Any serious Diagnostic and Statistical Manual axis II disorder	5 (6.7%)
Any mental disorder (nature/severity not specified)	4 (5.3%)
Any serious Diagnostic and Statistical Manual axis I disorder	3 (4.0%)

Note: 45 of the 75 (60%) trials in the 2020 Cochrane review of psychological treatments for chronic pain^118^ explicitly excluded people for at least one of the reasons listed above. Exclusion criteria of the primary studies were reviewed by the authors of the current topical review.

* % was computed from the total number of trials (n = 75). The mental health exclusion criteria are not mutually exclusive, and studies often reported multiple mental health exclusions, including for other mental health problems not classed as SMI.

† Some studies described the presence of these disorders without further qualification, whereas others qualified exclusion if the disorder was “poorly controlled or untreated.”

SMI, severe mental illness.

Pharmacological management of pain in SMI is complicated by the potential for harmful side effects and interactions with psychotropic medications and the underlying mental health condition.^50,65,85^ Antidepressants, including serotonin–norepinephrine reuptake inhibitors, are effective for pain management in the absence of depression^113^ and of course may improve comorbid depression^64^; however, unopposed antidepressants may destabilise mood in bipolar disorder. Collaborative pharmacological and psychological care for comorbid pain and major depression is promising, but scarce.^2^

The potential benefits and harms of other analgesics need careful consideration for people with SMI, as in the general population. In particular, people with major depression and bipolar disorder (but not schizophrenia^84^) are more likely than age- and sex-matched controls without mental illness to receive long-term opioids and experience adverse effects. Research is needed to determine whether these findings persist after reduced opioid prescribing in many countries. Cannabis and cannabinoids are increasingly discussed for pain despite evidence that cannabis use is associated with an increased risk of psychosis and increased risk of relapse and rehospitalization among people with psychosis.^43,72,99^ Guidelines advise against antipsychotics, gabapentinoids, benzodiazepines, and ketamine for chronic primary pain,^78^ but when used for psychiatric comorbidities,^8,98,100^ they may help comorbid pain. Recent advances in drugs targeting sleep disturbance^53,56^ and transcranial magnetic stimulation^62^ may prove fruitful for comorbid pain and SMI. There is a particular evidence gap for analgesics in people with bipolar disorder and psychosis, and research is thus needed. Medication optimisation for pain and SMI must occur alongside an interdisciplinary approach.^78^

## 3. Psychological and exercise-based interventions for severe mental illness in the wider context: synergies with pain management

For decades, pharmacological interventions were the main treatment offered for SMI. However, evidence for psychological treatments in psychosis, bipolar disorder, and severe depression has increased significantly. There has also been a growing focus on exercise-based interventions in this population.

### 3.1. Cognitive-behavioral treatments

Cognitive-behavioral therapy for psychosis, bipolar disorder, and depression uses cognitive and behavioral strategies to reduce distress and illness-related disabilities, facilitate adaptive coping, and support recovery goals. Individualised formulations of problem development, maintenance, and exacerbation are a central feature. Cognitive-behavioral therapy has efficacy for improving symptoms of psychosis, bipolar disorder, and severe depression.^20,83,101,115^ There is clear overlap in the use of cognitive-behavioral therapy for these disorders and evidence-based cognitive-behavioral methods for pain, including facilitating use of adaptive strategies to manage distressing thoughts and feelings and engage in personally meaningful activities in the presence of difficulties.^47,118^ This overlap can facilitate integration of cognitive-behavioral treatments for pain and SMI.

### 3.2. Family and carer interventions

Many people with SMI are closely supported by “informal” carers, primarily close relatives, whose support is key in determining outcomes.^79^ For those with family contact, family interventions are associated with reduced relapse and hospitalisation in people with psychosis and bipolar disorder.^12,18,87,90^ Family members often identify physical health problems and facilitate timely receipt of assessments and interventions.^82^ Therefore, family members are key in advocating for and supporting people with SMI to manage pain and its causes. It is also important to consider how certain caregiver behaviours and communication patterns, such as invalidating or overly solicitous responses toward pain expression may affect distress and disability in people with SMI and pain.^14^

Importantly, caregiving can adversely affect carer well-being. Carers of people with psychosis are less likely to continue caregiving when in poor health, hence the importance of identifying factors that adversely affect their health.^82^ Common mental disorders, sleep difficulties, and isolation are common in SMI carers.^60,102^ In addition, reports of pain may be elevated in carers of people with psychosis compared with noncarer peers, although the mechanisms underpinning this are unclear.^40^ Understanding how pain is experienced, communicated, and managed in SMI carers is important to develop tailored interventions. For example, delivering cognitive-behavioural approaches for pain jointly for people with SMI and their carers may enable both individuals to develop more adaptive responses to pain and create a healthier caregiving relationship. The need for a joint cognitive-behavioural approach has been discussed for couples affected by pain, but there is limited evidence to date.^13^

### 3.3. Exercise-based treatments

Persistent pain and SMI are, individually, associated with low physical activity.^105,112^ As it does in the general population, persistent pain also influences the ability of people with SMI to be active.^108,111^ Thus, despite the plethora of benefits of physical activity for physical health, pain, and mental health seen in meta-analyses of RCTs in persistent pain^33^ and SMI populations,^112^ both disorders are associated with underactivity.

Little is known about specific levels and benefits of physical activity for individuals with persistent pain and SMI. Exercise-based treatments hold great promise to improve health, well-being, and social connections in this group, as in the general population.^86^ Specifically, recent European^112^ and World Psychiatric Association guidelines identify that physical activity (including aerobic and resistance training) can reduce the risk for SMI onset and improve mental health symptoms, cognition, quality of life, and cardiorespiratory fitness in people with SMI.^31^ However, data on the impact of physical activity for pain in SMI are unclear, despite the positive effects noted in persistent pain generally.^33,97^

People with persistent pain and SMI experience a range of barriers to physical activity, including low mood and motivation, fatigue, isolation, lack of support, stigma, financial constraints, and service fragmentation.^29,71^ The multitude of barriers to and any facilitators of physical activity in people with SMI and persistent pain need to be better understood. This could help to develop or repurpose models of increased movement in this population. Historically, access to physical activity has been low, but the recent focus on improving the physical health, particularly metabolic health, of those with SMI has seen an increase,^59,71,93^ although effects are not well evaluated, particularly in the context of pain in this population.

## 4. Opportunities to improve integration of care for pain and severe mental illness

There is recognition of the need to better integrate physical and mental health services, with notable innovations to this end.^5,55,76^ At present, however, treatments for pain and SMI often occur in isolation within separate services and serially, despite clear synergies. Research is needed to understand how to optimise treatment integration and how to best adapt existing pain management pathways so that they are used by and work well for people with SMI. Opportunities to improve integration of care are briefly outlined.

### 4.1. Improving pain recognition and assessment

Identifying people with SMI who have or are at risk of experiencing pain is essential.^109^ Research should explore differences in pain communication between people with psychosis, bipolar disorder, and severe depression and healthcare professionals' sensitivity to that communication and how this might vary depending on patient racial and ethnic minority status. The latter point is particularly important as people from racial and ethnic minority groups are disproportionately diagnosed with SMI (eg, schizophrenia)^80^ and conditions where pain constitutes a primary component (eg, sickle cell) and experience higher healthcare inequalities.^45,80^ Pain assessment exclusively based on self-report may be challenging for people with psychosis who are less likely to self-identify pain and, depending on service setting, might be more inclined to underplay difficulties due to concerns of receiving additional services/treatments. Therefore, nonverbal assessment of pain behaviour is important.^41,42^ In addition, families or close friends can provide vital information given their critical caregiving role and should thus be included in the pain assessment process where possible. Nonverbal assessments of pain behaviours, such as facial expressions (eg, grimacing), body movements (eg, guarding), and interpersonal changes (eg, not wanting to be touched), are well-validated in other populations where self-report is problematic, such as dementia.^42^ Research is needed to determine the utility of these tools in people with psychosis.

Reports of severe pain in people with SMI may contribute to inappropriate medical management of pain, such as long-term opioid prescribing.^84^ Severe pain may prompt use of invasive treatments, which must be carefully considered because, for example, people with psychosis and bipolar disorder have an increased risk of infection and readmission after surgery for painful conditions compared with those without mental illness.^52^ Aggressive pain treatment may lead to underrecognition and treatment of SMI. Therefore, early detection of SMI in people with severe pain is also imperative to enable provision of appropriate treatment and reduce the risk of iatrogenic harm.

Despite increasing awareness and willingness to discuss mental health, SMI continues to be highly stigmatised,^74^ which may further impede pain recognition. Healthcare professionals underestimate pain in the presence of perceived “psychosocial” problems,^22^ making discounting of pain in people with SMI particularly likely. Indeed, there is evidence that they experience diagnostic overshadowing for physical health care.^30^ In addition to limiting treatment access, pain-related invalidation, stigma, and discrimination exacerbate distress.^94–96^ Investigation is needed to understand the impact of intersecting experiences of stigma and discrimination in people with SMI and pain and how to address these. At the structural level,^104^ for example, service planning and funding that enable integration of treatments for pain and SMI may reduce stigma and discrimination experienced when fragmented services exclude people.^71^ At the interpersonal level, role plays developed with people with lived experience may be useful for training to improve clinicians' communication with people with pain and SMI so that interactions are empathic and respectful.^25,104^ At the individual level, psychological interventions may help people respond effectively to the personal impacts of stigma and discrimination, although effects may be modest in the absence of intervention at the other levels.^96,104^

### 4.2. Stakeholder involvement

Meaningful involvement of people with lived experience of SMI and pain will be crucial^7,11,81,91^ to improve existing pain management pathways and develop integrated treatments. Involvement of a range of stakeholders is also needed, including carers, mental health and pain management clinicians from primary to tertiary care, healthcare commissioners, policymakers, and third sector organisations. Stakeholder involvement can include, for example, Priority Setting Partnerships, which can be modelled after exemplary work in paediatric pain.^9^ Stakeholders can provide crucial input to shape pain assessment and intervention tailoring, identify meaningful treatment targets, optimize pathways into treatment, and enhance clinician training. Drawing again from work in paediatric pain,^15^ stakeholders can shape dissemination and implementation of knowledge about research and best practice.

### 4.3. Treating and evaluating the individual

To rapidly advance the understanding of pain management in people with SMI, innovations in treatment evaluation are needed. RCTs are the gold standard for evaluating interventions but require highly selected samples, protocol-adherent treatment delivery, and group-based analysis.^75^ This limits their potential to inform flexible treatment delivery that addresses the context and complexity of individuals in practice.^116^

Single-case experimental designs (SCEDs) are well suited to advance development of integrated treatments in a manner that appreciates the heterogeneity of diagnostically specific challenges relating to pain and SMI. SCEDs are a rigorous alternative to RCTs that can enable personalized care.^17,61,63,68,116^ In SCEDs, the individual is their own control through intensive, repeated measurement during baseline and treatment phases.^68,75^ Multiple SCEDs can be undertaken informing multiple treatment approaches with heterogeneous participants using fewer resources than would a single RCT with a homogenous sample.^68,75^ The frequency of assessments in SCEDs provides greater opportunity than that in RCTs to evaluate treatment mechanisms necessary to improve effectiveness.^10^

Single-case experimental designs are not without limitations. The baseline phase can be demanding and is not feasible when urgent treatment is needed, such as for active suicidality.^54^ The generalizability of SCEDs has been questioned, but replication across patients and settings allows generalizability to be tested.^54^ Although RCTs offer many advantages, SCEDs are highlighted here as a lesser used methodology with underexplored possibilities for developing and evaluating treatments in people with pain and SMI.

### 4.4. Implementation science

Implementation science holds promise to ensure that research into new models of care has a real-world impact. Implementation science is “the scientific study of methods to promote the systematic uptake of research findings and other evidence-based practices into routine practice, and hence, to improve the quality and effectiveness of health services and care”^24^(p. 1). Implementation frameworks draw on behavioral and social science and identify individual-level (eg, clinician knowledge, motivation, and professional role/identity) and structural-level (eg, resources, organizational culture, and policy) factors that influence intervention uptake.^4,15,44,57,66^ The number and complexity of available theories can present a challenge for implementation research, particularly where empirical findings are underused in refining theory.^57^

Nonetheless, implementation theories may help identify barriers to and facilitators of integrating existing and novel treatments for pain and SMI in practice. For example, quantitative and qualitative methods can investigate knowledge, skill, and perceptions about professional roles for pain/SMI treatment among clinicians in pain and mental health services. This could inform novel training and supervision models, which could be developed using implementation principles to ensure uptake, scalability, and sustainability.^38^ In addition, linking existing data sets of, for example, referral patterns and treatment outcomes from primary to tertiary care can provide insights into opportunities for integration across the system. There are emerging local examples of how rapid communication between mental and physical health services can improve outcomes for people with SMI.^69^

As an example of bringing different methods together, data set linkages may identify repeat referrals of patients between pain and mental health services. Interviews with mental health clinicians may reveal a need for training in pain management, whereas interviews with pain clinicians may reveal perceptions that the format (eg, group-based) or duration (eg, number of sessions) of commissioned treatment is unsuitable for people with pain and SMI. Together, these data could argue for more collaboration and mutual training between pain and mental health clinicians and coproduction with service users to better deliver holistic pain management within their services. Surveys of service funders could identify key targets (eg, reduced referrals and improved patient satisfaction) that would allow this model to be sustainably funded.

To conclude, there is an urgent need to advance research and practice to improve pain management in people with SMI. This work should draw on synergies in the existing evidence for managing pain and SMI. Meaningful involvement of people with lived experience is essential to advance this agenda.

## Supplemental video content

A video abstract associated with this article can be found at http://links.lww.com/PAIN/B605.
